# *MetaLonDA:* a flexible R package for identifying time intervals of differentially abundant features in metagenomic longitudinal studies

**DOI:** 10.1186/s40168-018-0402-y

**Published:** 2018-02-13

**Authors:** Ahmed A. Metwally, Jie Yang, Christian Ascoli, Yang Dai, Patricia W. Finn, David L. Perkins

**Affiliations:** 1 Department of Bioengineering, University of Illinois at Chicago, Chicago, 60607 IL USA; 2 Department of Medicine, University of Illinois at Chicago, Chicago, 60612 IL USA; 3 Department of Computer Science, University of Illinois at Chicago, Chicago, 60607 IL USA; 4 Department of Mathematics, Statistics, and Computer Science, University of Illinois at Chicago, Chicago, 60607 IL USA; 5 Department of Microbiology and Immunology, University of Illinois at Chicago, Chicago, 60612 IL USA; 6 Department of Surgery, University of Illinois at Chicago, Chicago, 60612 IL USA

**Keywords:** Metagenomics, Microbiome, Differential abundance, Longitudinal studies, Time series, Smoothing splines, Negative binomial distribution

## Abstract

**Background:**

Microbial longitudinal studies are powerful experimental designs utilized to classify diseases, determine prognosis, and analyze microbial systems dynamics. In longitudinal studies, only identifying differential features between two phenotypes does not provide sufficient information to determine whether a change in the relative abundance is short-term or continuous. Furthermore, sample collection in longitudinal studies suffers from all forms of variability such as a different number of subjects per phenotypic group, a different number of samples per subject, and samples not collected at consistent time points. These inconsistencies are common in studies that collect samples from human subjects.

**Results:**

We present *MetaLonDA*, an R package that is capable of identifying significant time intervals of differentially abundant microbial features. *MetaLonDA* is flexible such that it can perform differential abundance tests despite inconsistencies associated with sample collection. Extensive experiments on simulated datasets quantitatively demonstrate the effectiveness of *MetaLonDA* with significant improvement over alternative methods. We applied *MetaLonDA* to the DIABIMMUNE cohort (https://pubs.broadinstitute.org/diabimmune) substantiating significant early lifetime intervals of exposure to *Bacteroides* and *Bifidobacterium* in Finnish and Russian infants. Additionally, we established significant time intervals during which novel differentially relative abundant microbial genera may contribute to aberrant immunogenicity and development of autoimmune disease.

**Conclusion:**

*MetaLonDA* is computationally efficient and can be run on desktop machines. The identified differentially abundant features and their time intervals have the potential to distinguish microbial biomarkers that may be used for microbial reconstitution through bacteriotherapy, probiotics, or antibiotics. Moreover, *MetaLonDA* can be applied to any longitudinal count data such as metagenomic sequencing, 16S rRNA gene sequencing, or RNAseq. *MetaLonDA* is publicly available on CRAN (https://CRAN.R-project.org/package=MetaLonDA).

**Electronic supplementary material:**

The online version of this article (10.1186/s40168-018-0402-y) contains supplementary material, which is available to authorized users.

## Background

Longitudinal studies of the microbiome have gained tremendous popularity during the past few years due to the ability to detect trends of microbiome changes over time and relate these changes to disease progression in different parts of the body, such as the gut, kidney, skin, or lung [1–6]. In addition, there has been a drastic reduction in sequencing cost that has made longitudinal studies more affordable on a large scale.

Two major types of analysis can be performed in longitudinal microbial studies that snapshot studies cannot provide: (a) analysis over time to capture the dynamics of microbial interactions [7, 8] and (b) association studies that correlate change of microbial features, such as taxonomies, genes, or average relative abundance of pathway components, with a phenotypic group. The latter analysis is usually challenged by variability in longitudinal sample collections, including inconsistencies in the number of subjects per phenotype, number of samples per subject, and sample collection at inconsistent time points. These inconsistencies increase with the level of difficulty with which samples are obtained from the subjects. For example, in humans, the variability decreases in samples collected non-invasively (e.g., stool and urine samples) but increases in the invasive procedures (e.g., bronchoalveolar lavage (BAL) samples which are extracted from the lung by bronchoscopy).

One solution to address this variability is to bin samples into a certain number of windows between the start and end times of the study course by selecting the nearest sample in time for each bin [2], then, compare the microbial feature’s relative abundance or diversity indices [9–11] between any pair of time points to characterize any pairwise changes. The limitation of this approach is that it deals with the longitudinal data points as a collection of static snapshots and ignores temporal dependencies. Furthermore, if more than one sample is taken in the same time window, it may result in either retaining only one sample and excluding the others or taking the average of the measured feature’s values, which may lead to mischaracterizing the exact microbial behavior.

Another strategy is to identify time intervals of differentially abundant microbial features. To date, two methods have been proposed: the first is *MetaSplines* [12], and the second is *MetaDprof* [13]. *MetaSplines* and *MetaDprof* are both based on the Gaussian smoothing spline ANOVA (SS-ANOVA) approach [14–16], where the Gaussian distribution is used to model the number of reads mapped to each microbial feature. *MetaSplines* has a higher sensitivity of detecting time intervals of differentially abundant features than *MetaDprof*, but *MetaDprof* has higher specificity [13]. *MetaDprof* has a major drawback, namely, its implementation assumes consistency in longitudinal microbial samples, such that it is only able to perform the analysis on an equivalent number of subjects per phenotypic group, the same number of samples from each subject, and the same elapsed time between adjacent time points. However, these conditions are rarely fulfilled in human microbiome longitudinal studies.

In this paper, we introduce *MetaLonDA* (Metagenomic Longitudinal Differential Abundance method), an R package that performs longitudinal differential abundance tests in a strategy that can identify time intervals of microbial features that are significantly over/under abundant in a phenotypic group. *MetaLonDA* is flexible such that it can handle all types of inconsistencies in microbial sample collections. The identified differentially abundant features and their time intervals have the potential to distinguish microbial biomarkers that may be used for microbial reconstitution therapy through bacteriotherapy, probiotics, or antibiotics and may also suggest timing and duration of the therapy.

## Implementation

**Fig. 1:**
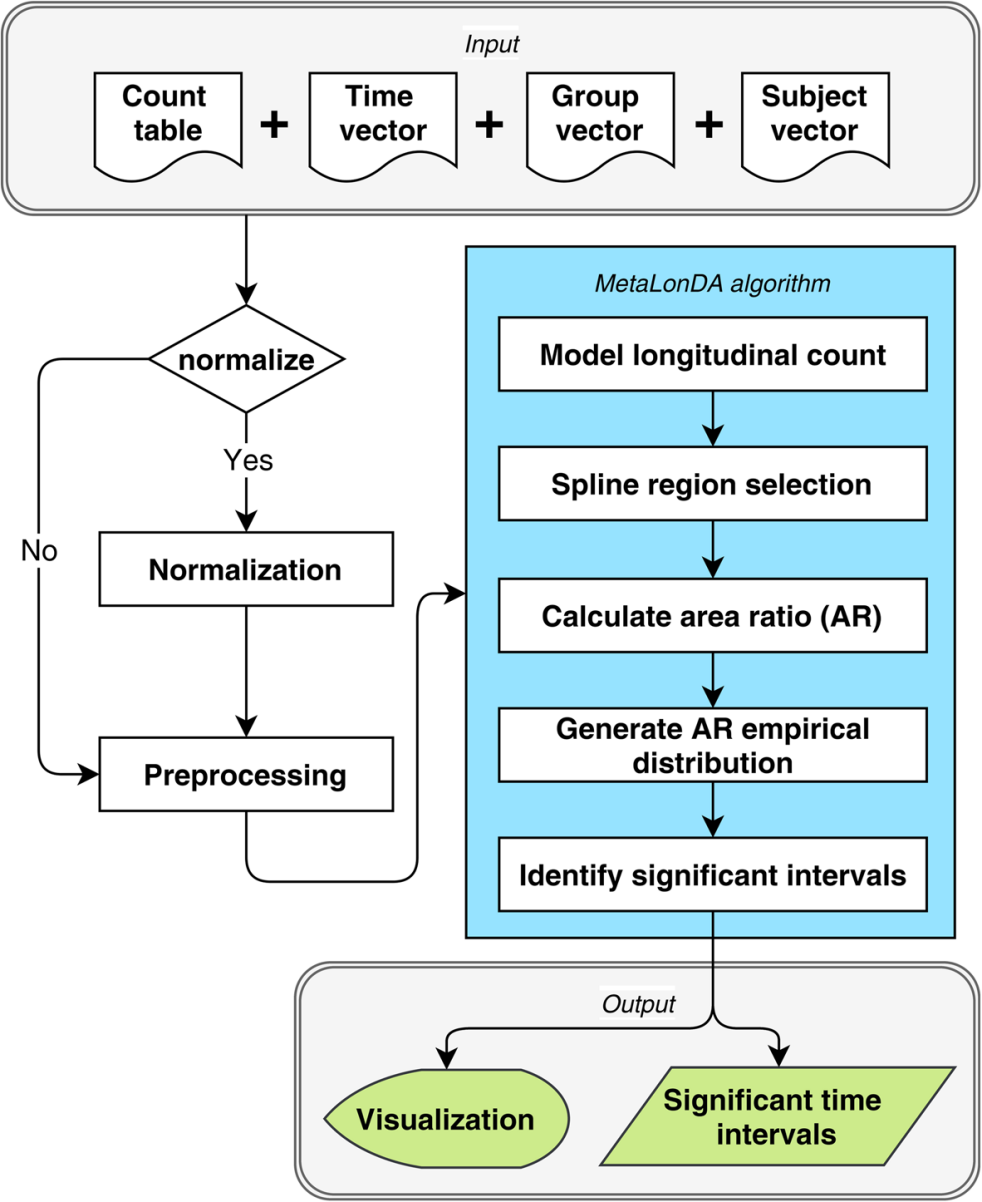
*MetaLonDA* framework

The main components of the *MetaLonDA* framework are shown in Fig. 1.

### Input

Metagenomic reads are processed for each sample to construct taxonomic and/or functional profiles [17–20]. The taxonomic profiles, functional profiles, or both for all samples from different subjects are then integrated into one count table *C* with a dimension of *m* × *n*, where *m* denotes the number of microbial features and *n* denotes the number of metagenomic samples. *C*(*i*,*j*) represents the number of reads from sample *j* that mapped to microbial feature *i*. The count table *C* is the main input to *MetaLonDA*. Additionally, three vectors each of length *n* are needed for *MetaLonDA* to perform the analysis: (a) time of sample collection vector *T*, (b) phenotypic group vector *G*, and (c) subject ID vector *I*. As previously highlighted, *MetaLonDA* supports unequal numbers of samples between subjects, unequal numbers of subjects between phenotypic groups, and uneven elapsed time between time points.

### Normalization

Since metagenomic samples may have different sequencing depths, the aggregated metagenomic counts need to be normalized among samples [21, 22]. *MetaLonDA* incorporates three different normalization methods into its framework: (a) cumulative sum scaling [12], (b) median-of-ratios scaling factor [23], and (c) trimmed mean of *M* values [24]. If the count table is already normalized, the normalization step should be skipped in *MetaLonDA*. As a preprocessing step for *MetaLonDA* and based on a user-specified threshold, relatively low abundant features are removed from the metagenomic count table. In our model, we assume that the normalized counts of each feature follow a negative binomial (NB) distribution, which is different from modeling the original counts as NB distributed after incorporating a size factor into the mean as in *DESeq2* [23].

### *MetaLonDA* core algorithm

The *MetaLonDA* algorithm relies on two modeling components: the NB distribution for modeling the mapped read counts for each feature and the semi-parametric SS-ANOVA technique for modeling longitudinal profiles associated with each phenotype [25]. By fixing a feature *f*=1,…,*F*, the data under consideration are the random variables *Y*_*tki*_ or their observations *y*_*tki*_ of mapped reads of the *i*^*th*^ subject of phenotype *k* to the feature *f* at time point *t*, where *t*=1,…,*T*, *k*=1,2, and subject *i*=1,…,*n*_*k*_. The random variable *Y*_*tki*_ is assumed to follow NB distribution as shown in Eq. (1), with integer *α*>0 and success probability *p*(*t*,*k*)∈(0,1).

1 $$  Y_{tki} \sim \text{NB}(\alpha, p(t, k))  $$

Assuming *Y*_*tki*_’s are independent, the log-likelihood given time-course metagenomic count profiles $\phantom {\dot {i}\!}{y} = \{y_{tki}\}_{t = 1,\ldots,T; k = 1,2; i = 1, \ldots, n_{k}}$ is calculated as in Eq. (2)

2 $$ \mathcal{L} = \log L({p}, \alpha \mid {Y}={y})  $$

We seek the estimation of model parameters *α* and *p*(*t*,*k*) by maximizing Eq. (2) (Additional file 1). To model the time and phenotypic effect, we use a general linear model with a logit link as in Eq. (3)

3 $$  \eta (t, k)= \log \frac{p(t, k)} {1-p(t, k)}  $$

Following [16], in order to control the smoothness of the function *η*, a roughness penalty *J*(*η*) is added to the minus log-likelihood together with the smoothing parameter *λ*>0 for the trade-off between the goodness of fit and the smoothness of the spline curve as in Eq. (4), where the smoothing parameter *λ* is determined by cross-validation procedure.

4 $$  \min_{p,\alpha} {-\mathcal{L} + \lambda\cdot J(\eta)}  $$

The solution to the optimization problem in Eq. (4) leads to a smoothing spline that fits the reads from samples across multiple time points. After fitting longitudinal profiles in each phenotypic group with a NB smoothing spline, the area ratio AR_*t*,*t*+1_ between the two modeled curves per unit time interval is calculated as in Eq. (5), where $A_{t,t+1}^{k_{1}}$ and $A_{t,t+1}^{k_{2}}$ denote the area under the spline curve from time *t* to time *t* + 1 for group 1 and group 2, respectively, *t*=1,…,*T*−1.

5 $$  \text{AR}_{t,t+1}= \frac{A_{t,t+1}^{k_{1}} - A_{t,t+1}^{k_{2}}}{\text{max}(A_{t,t+1}^{k_{1}}, A_{t,t+1}^{k_{2}})}  $$

The *p*-value of each time interval is then calculated based on the AR_*t*,*t*+1_ empirical distribution which is constructed by a permutation test. The significant time intervals are identified as those with *p*-value < threshold (default = 0.05) after multiple testing corrections using Benjamini-Hochberg (BH) [26]. The complete mathematical derivation of the *MetaLonDA* algorithm is illustrated in details in Additional file 1.

### Output format and visualization

*MetaLonDA* outputs a table that includes significant features, start and end points of the corresponding significant intervals, the adjusted *p*-value of each significant time interval, and the phenotypic group in which the corresponding feature is more abundant. In addition to the output table, *MetaLonDA* produces two types of visualizations: (a) a figure showing the fitted splines of each group and the associated time interval for each feature that has at least one significant time interval and (b) a figure visualizing the identified time intervals of the differentially abundant features (as shown in Fig. 6).

## Results and discussion

### Evaluation of the negative binomial assumption

**Fig. 2:**
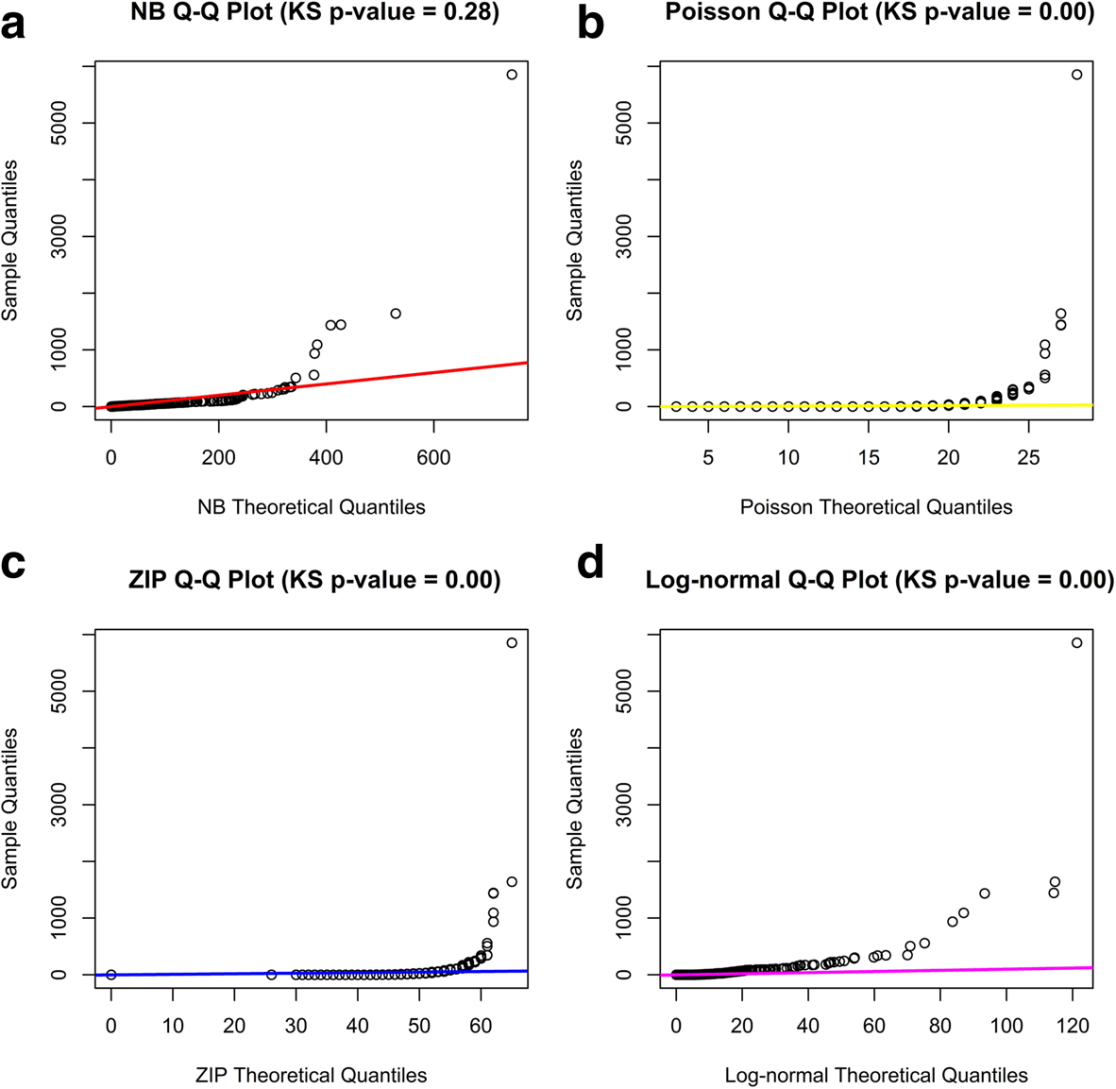
Quantile-quantile plot between different theoretical distributions and *Klebsiella* read counts. Each sub-figure represents a different distribution: (**a**) NB distribution, (**b**) Poisson distribution, (**c**) ZIP distribution, (**d**) lognormal distribution. The *p*-value above each sub-figure represents the significance of the KS test between the sample quantiles and the theoretical quantiles of the corresponding distribution. The NB distribution is most appropriate to model the OTU count among other standard distributions

One major assumption of *MetaLonDA* is that the number of metagenomic reads mapped to microbial features follows a NB distribution. To evaluate this assumption, we extracted the count data from Caporaso et al. [1]. In this dataset, microbial samples were taken on a daily basis from a man and a woman over a period of 15 and 6 months, respectively, from four different body sites. The obtained read counts were normalized using the median-of-ratios scaling factor method [23]. After filtering out the relatively rare operational taxonomic units (OTUs) with fewer than five reads, a total of 750 OTUs were selected from 1967 samples. The Q-Q plot in Fig. 2 exemplifies the suitability of modeling read counts of *Klebsiella* species using different parametric distributions, namely, NB, Poisson, zero-inflated Poisson (ZIP), and lognormal distributions. The theoretical quantiles of each parametric distribution are calculated from random numbers generated from each parametric distribution with parameters estimated from each OTU read count (parameter fitting methods for each distribution are discussed in Additional file 1). The *p*-value on the top of each sub-figure of Fig. 2 represents the BH-adjusted *p*-value of the two-sample Kolmogorov-Smirnov (KS) test [27], where a higher *p*-value indicates that the two samples are derived from the same population distribution and smaller *p*-value indicates that the two samples are drawn from different population distributions. In the case of *Klebsiella*, only the NB distribution is considered suitable (*p*-value =0.28).

To evaluate all other features, we applied the KS test to the read counts of each of the 750 OTUs and the sampled numbers from the corresponding parametric statistical distribution that had the same parameters as estimated from the read counts. Table 1 summarizes the number of features that do not show significant divergence (*p*-value > 0.05 after BH multiple testing corrections) with NB, ZIP, Poisson, lognormal, exponential, half-normal, and normal distributions. Out of the 750 features, 96% were modeled appropriately using NB distribution. In comparison, ZIP and Poisson were appropriate for 41% and 26% of the OTUs, respectively, whereas the rest of the parametric distributions employed in this analysis barely fit. This indicates the appropriate use of NB as a parametric distribution model for *MetaLonDA* when compared to other standard parametric distributions. Furthermore, this finding is consistent with previous studies that show that cross-sectional differential abundance methods that use a NB distribution to model microbial features outperform methods that rely on other distributions, especially when the number of samples is small [28].

**Table 1:** Number and percentage of species out of 750 species that do not show significant differences (KS *p*-value > 0.05) with various standard statistical parametric distributions

	Number	Percentage
NB	721	96.13
ZIP	309	41.20
Poisson	201	26.80
Lognormal	1	0.13
Exponential	0	0
Half-normal	0	0
Normal	0	0

### Performance evaluation based on simulated datasets

**Fig. 3:**
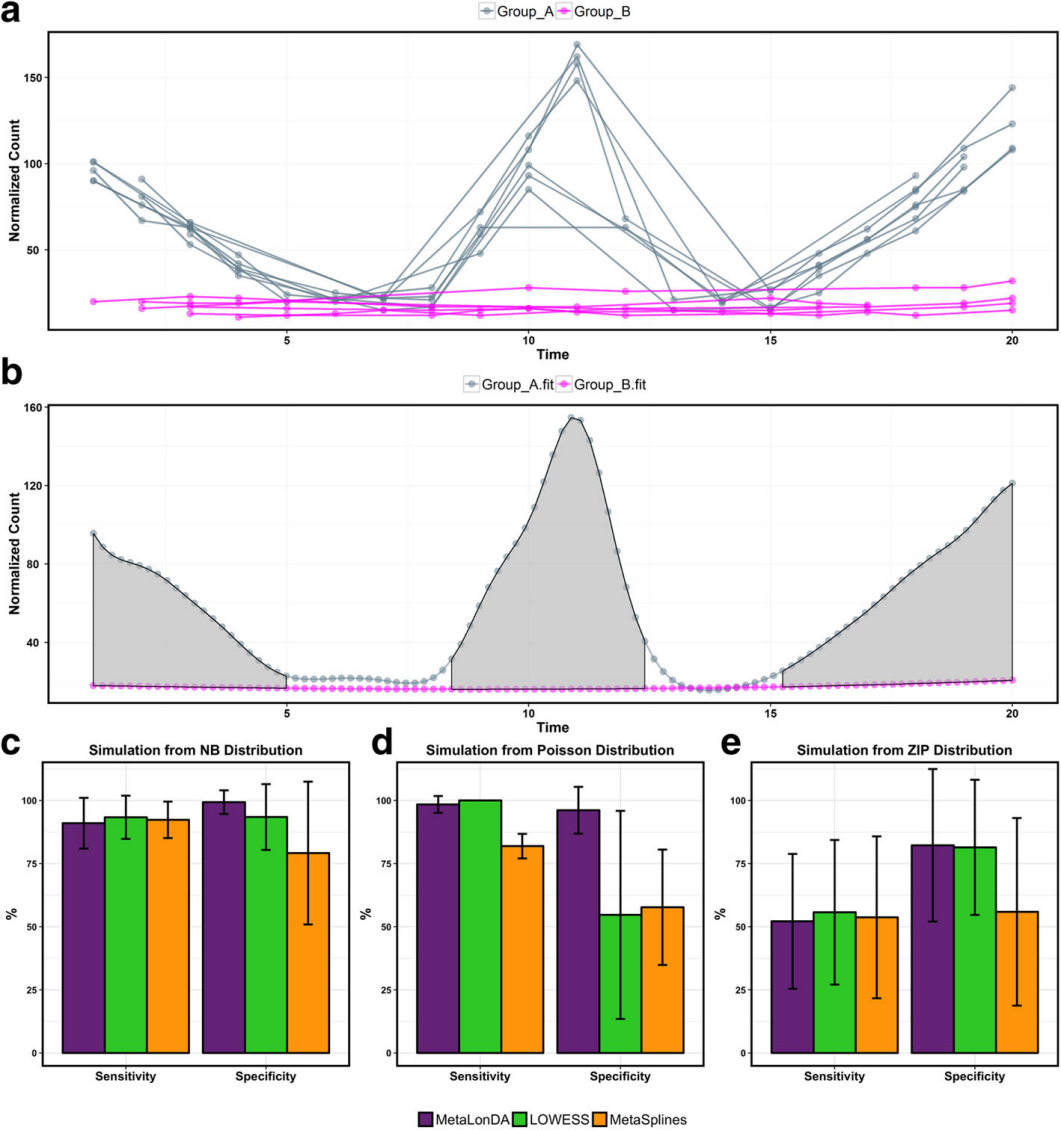
Pattern and performance evaluation of data simulated from various statistical distributions. (**a**) The pattern of the simulated longitudinal features. Each differentially abundant feature has time intervals between group A and B at [1,5] $\bigcup $ [8,13] $\bigcup $ [15,20] and non-differential time intervals [5,8] $\bigcup $ [13,15]. The simulated data mimics inconsistencies in sample collection (different number of subjects per group, different number of samples per subject, and samples not equally spaced.) (**b**) The fitted smoothing spline of each group and the highlighted significant time intervals between the two groups. (**c**–**e**) The performance of different tools using data simulated from NB, Poisson, and zero-inflated Poisson, respectively. Each bar represents the mean among 1000 features, and the error bar represents the standard deviation. *MetaLonDA* always has a higher specificity than *LOWESS* and *MetaSplines*. This shows *MetaLonDA*’s robustness among different distributions

In order to benchmark *MetaLonDA*’s performance, we performed a comprehensive simulation study. Longitudinal features (*n* = 1000) were simulated from NB, Poisson, and ZIP distributions using the *corcounts* R package [29]. Although read counts of metagenomic features follow NB distribution as shown in Table 1, the purpose of simulating data from Poisson and ZIP was to evaluate the robustness of *MetaLonDA* when read counts fail to follow the NB distribution. These simulated features were categorized into two types: (a) 500 differentially abundant features between the two testing groups and (b) 500 features that were not differentially abundant between the two testing groups. In the case of the differentially abundant features (demonstrated in Fig. 3a), the mean *μ*(*t*) pattern is simulated to be differentially abundant in three regions: (a) at the start of the study course, (b) at the end of the study course, and (c) in the middle of the study course (Additional file 1). In the case of non-differentially abundant features, the  where  denotes normal distribution and *t* = 0,…,20.

For features simulated from the NB distribution, we used a size factor equal to 40/*μ*(*t*). In the case of Poisson distribution, we used *λ* = *μ*(*t*), and in the case of zero-inflated Poisson distribution, we used *p*(*y*=0)=0.3 for the zero-inflation parameter. Our choice of the zero-inflation probability was based on the analysis of $\hat {p}(y=0)$ when we fitted all features in the Caporaso et al., study [1] with the ZIP distribution (Table 1). The histogram in Additional file 2 shows that 75% of the $\hat {p}(y\ =\ 0)$ is less than 0.3 (median of $\hat {p}$ = 0.1). Therefore, our choice of 0.3 is to evaluate how *MetaLonDA* performs in this case of simulated zero inflation.

In order to mimic the correlation behavior between adjacent time points in longitudinal studies, the simulation of read counts of adjacent samples followed the first-order autoregressive model [30] with a correlation coefficient *ρ* = 0.9. Datasets were simulated for 15 subjects with 20 time points each (*T* = 20). Additionally, to mimic inconsistencies in the number of subjects per group and number of samples per subject, we randomly chose 11 samples from 8 subjects from group A and 8 samples from 6 subjects from group B (Fig. 3a).

We proceeded to evaluate the performance of *MetaLonDA* in comparison to *MetaSplines*, *MetaDprof*, and *LOWESS* [31]. *LOWESS* is a non-parametric local regression model that is based on combining multiple regression models in a *k*-nearest-neighbor-based meta-model. In the context of this paper, *LOWESS* refers to using the *LOWESS* regression model to substitute the NB distribution in *MetaLonDA*’s framework. Each method was run for 1000 permutations to construct the AR empirical distribution. The *p*-value threshold was set to 0.05 after multiple testing corrections using BH. The rest of the parameters were set to default. The assessment is based on the $\text {sensitivity} = \frac {TP}{TP+FN}$ and $\text {specificity} = \frac {TN}{TN+FP}$. In this context, *TP* represents the number of truly identified time intervals of differentially abundant features. *TN* represents the number of truly identified time intervals of non-differentially abundant features, *FP* represents the falsely identified time intervals of non-differentially abundant features, and *FN* represents the falsely identified time intervals of differentially abundant features.

Table 2 shows the performance evaluation based on consistent sampling, i.e., the ideal scenario which is rare. *MetaLonDA* has the most balanced prediction in terms of sensitivity and specificity followed by *MetaDprof* and *MetaSplines*.

**Table 2:** Performance evaluation of data simulated from various statistical distributions mimicking consistent sampling

	NB		Poisson		ZIP	
	Sensitivity (%)	Specificity (%)	Sensitivity (%)	Specificity (%)	Sensitivity (%)	Specificity (%)
*MetaLonDA*	98	95	99	96	84	90
*MetaDprof*	94	94	86	94	87	96
*LOWESS*	96	80	100	47	94	60
*MetaSplines*	81	79	85	59	60	64

Next, we benchmarked *MetaLonDA* using the inconsistent sampling scenario. In this experiment, *MetaDprof* was excluded since its package cannot handle the sampling inconsistencies. In the case of data simulated from NB distribution, Fig. 3c shows that *MetaLonDA* outperforms *MetaSplines* and *LOWESS* in sensitivity and specificity. On the other hand, in the case of data simulated from Poisson distribution, Fig. 3d demonstrates that *LOWESS* has a slightly better sensitivity than *MetaLonDA* (100 vs. 98%). But, the specificity of *LOWESS* and *MetaSplines* is very low when compared to *MetaLonDA* (50 vs. 95%). This is because *LOWESS* and *MetaSplines* over-fit the data. Lastly for the case of the zero-inflated Poisson, Fig. 3e shows that *MetaLonDA*, *MetaSplines*, and *LOWESS* have a comparatively low level of sensitivity(∼ 50%), but *MetaLonDA* has higher specificity. The reason behind this low sensitivity is the high zero inflation probability we chose for ZIP, *p*(*y* = 0) = 0.3. To summarize, *MetaLonDA* always maintains a very high specificity, in contrast to *LOWESS* and *MetaSplines*.

The execution time of *MetaLonDA*, *MetaDprof*, and *MetaSplines* is comparable and depends on the number of permutations used. Analysis of the simulated dataset from a NB distribution with 1000 features took 104 min with *MetaLonDA*, 113 min with *MetaDprof*, and 99 min with *MetaSplines*. The analysis was conducted on a MAC machine with 2.5 GHz Intel Core i7 processor and 16 GB 1600 MHz RAM. For the same analysis, *LOWESS* was slightly faster (87 min) because it does not have the complex smoothing spline optimization Eq. (4) that needs to be solved numerically.

### Performance evaluation on a biological dataset: hygiene hypothesis study

**Fig. 4:**
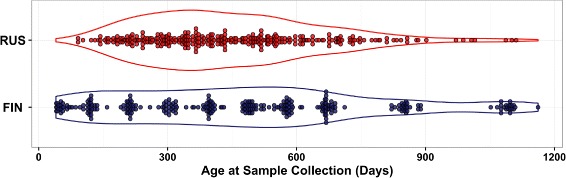
Time distribution of 585 stool samples (304 from 70 Russian and 281 from 71 Finnish) sequenced using MGS in the DIABIMMUNE project. The collected samples have various forms of inconsistencies, different numbers of subjects per group (70 Russian vs 71 Finnish infants), and different numbers of samples per subject (min = 1, max = 13), and the samples’ time points are not equally spaced

In order to assess the biological significance of the identified time intervals of differentially abundant features, we used a publicly available dataset from a longitudinal metagenomic study that investigates the hygiene hypothesis [3]. The study was part of the DIABIMMUNE project (https://pubs.broadinstitute.org/diabimmune). Stool samples were collected from 222 infants (74 from Russia, 74 from Finland, and 74 from Estonia) from birth to ∼ 3 years of age. In our analysis, we identified the time intervals with differentially abundant genera in Russian and Finnish infant guts. We focused on the 585 samples (304 from 70 Russian infants and 281 from 71 Finnish infants) that had been sequenced using metagenomic shotgun (MGS) sequencing. Figure 4 shows the distribution of time points of the stool samples collected from each group (Additional file 3 shows the distribution of time points per subject). Reads from the 585 sequenced samples were quality-controlled by filtering out low-quality reads, short reads (< 60 bp), and human reads. Taxonomic profiles were constructed using *MetaPhlAn2* [32]. The number of reads mapped to each taxonomic feature was then normalized to the reads per kilo-base per million (RPKM) sample reads to correct for bias due to differences in genome size and sequencing depth. The aggregated taxonomic profiles of all 585 samples revealed 128 genera.

In order to evaluate the suitability of using NB to model genera read counts before applying *MetaLonDA*, we conducted an analysis similar to the one shown in Table 1. We found that NB can be considered a good fit for 79% of the 128 genera (Additional file 4 shows a detailed comparison between different parametric distributions).

**Fig. 5:**
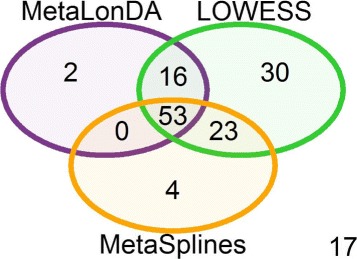
Number of genera identified as differentially abundant between the Finnish and Russian infants. Fifty-three common genera were identified as differentially abundant using the three tools. The “17” on the lower right corner represents the number of genera that were not identified at any time interval by *MetaLonDA*, *LOWESS*, or *MetaSplines*

**Fig. 6:**
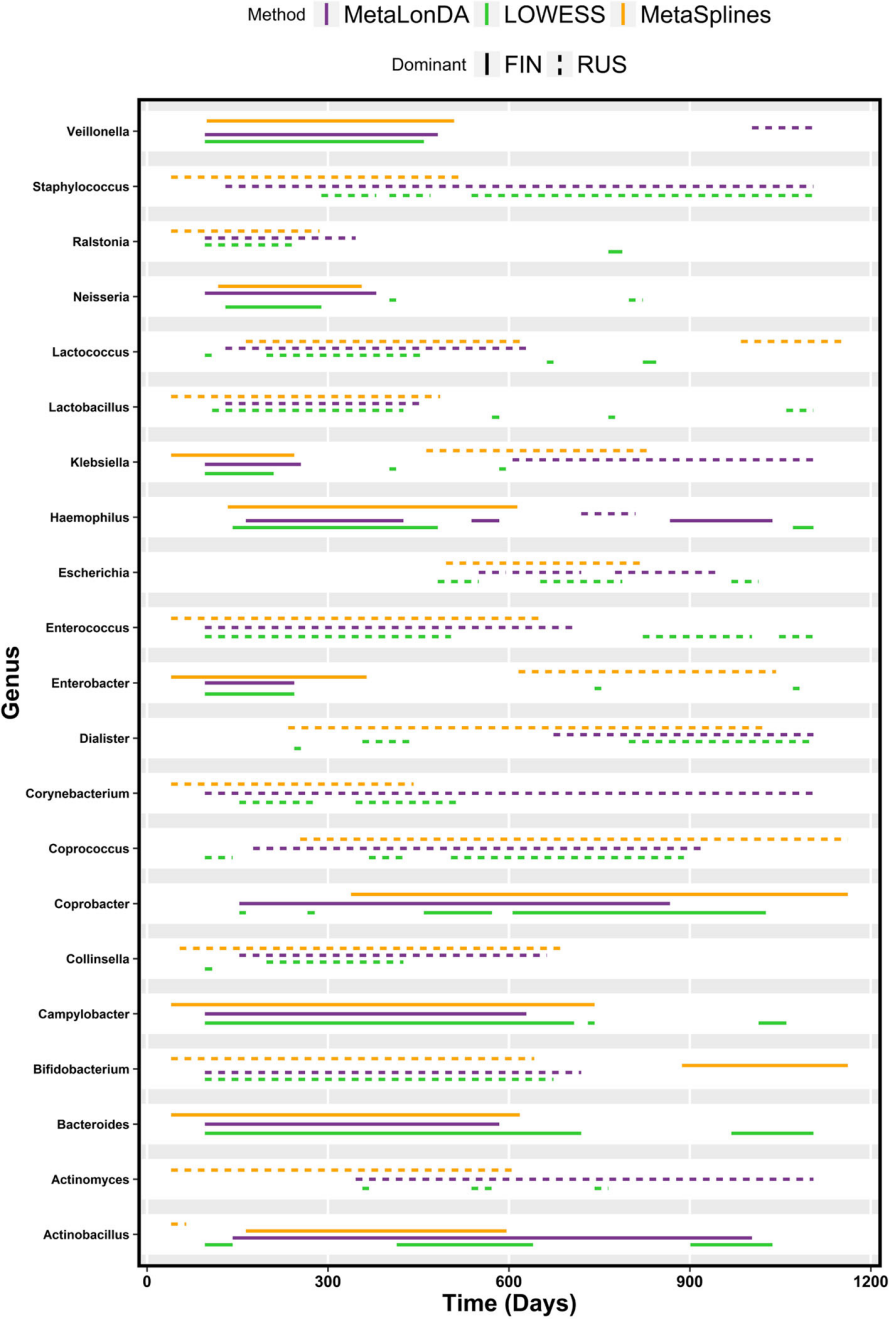
The time intervals of the mutually differentially abundant genera from Finnish and Russian infants identified by *MetaLonDA*, *LOWESS*, and *MetaSplines*. Each line represents significant time interval of the corresponding genera. *MetaLonDA* (purple), *LOWESS* (green), *MetaSplines* (orange). The solid lines represent the intervals where samples from the Finnish group have more reads, while the dashed lines represent the differential abundance intervals where samples from the Russian group have more reads

We applied *MetaLonDA*, *LOWESS*, and *MetaSplines* to identify the time intervals of the differentially abundant genera. We set the number of permutations for all three methods to 1000, *p*-value threshold =0.05, multiple testing correction method to BH, and other parameters to default. *MetaLonDA* identified 71 genera that have at least one time interval with differentially abundant genera, *LOWESS* identified 122 genera, and *MetaSplines* identified 80 genera. Although there are 53 mutually inclusive common genera identified by the three methods as shown in Fig. 5, this does not necessarily indicate that they share the same identified time intervals as demonstrated in Fig. 6. *LOWESS* identified 30 genera that neither *MetaSplines* nor *MetaLonDA* reported. Whereas *MetaLonDA* identified 2 genera that were not reported by either *LOWESS* or *MetaSplines*. These results emphasize the high control of false positive identifications by *MetaLonDA*. The previously discussed simulation study concluded that *LOWESS* and *MetaSplines* have lower specificity compared to *MetaLonDA*. Thus, *MetaLonDA* discovery of few significant time intervals is directly related to its increased specificity compared to the other two methods.

Figure 6 visualizes differences between the time intervals identified by *MetaLonDA*, *LOWESS*, and *MetaSplines* correlating with the major shared genera. In most cases, the time intervals identified by *MetaLonDA* were also identified by either *LOWESS*, *MetaSplines*, or both. One critical observation that likely contributes the greater number of false positives observed in *MetaSplines* is that it sometimes identifies time intervals where samples from one group are missing. The absence of one group’s samples can make the spline fitting uncontrollable [16]. For example, *MetaSplines* identified *Actinobacillus* as relatively more abundant in the Russian infants from day 40 until day 65, although the first Russian sample was collected 96 days after birth. *MetaLonDA* handles this situation by only reporting significant intervals during the time period when samples from all study groups are available. In the case of the hygiene hypothesis study, individual genera’s time intervals identified by *MetaLonDA* are bounded in the range of 96 to 1105 days. Day 96 was the day on which the first sample from a Russian infant was collected, and day 1105 is when the last Russian sample was collected (the first sample from Finnish infants was on day 41, and the last was on day 1162). Since we implemented *LOWESS* on the same *MetaLonDA* framework, it also handles this edge problem. A list of all time intervals identified by *MetaLonDA*, *LOWESS*, and *MetaSplines* are shown in Additional file 5. Additional file 6 shows the time intervals of differentially abundant genera identified by *MetaLonDA*, *LOWESS*, and *MetaSplines*, while Additional file 7 shows time intervals identified by *MetaLonDA* only.

In our analysis, *MetaLonDA* confirms the report by Vatanen et al. demonstrating that the genus *Bacteroides* is relatively more abundant during early time points in the Finnish group, whereas the genus *Bifidobacterium* is relatively more abundant in the Russian group [3]. *MetaLonDA* specifies that *Bacteroides* were significantly abundant during days 96–584 in Finnish infants, and *Bifidobacterium* were relatively more abundant in Russian infants from day 96 to day 720. Furthermore, in their study, Vatanen et al. noted that early life exposure to specific structurally distinct bacterial *lipopolysaccharides* (LPS) influences the development of autoimmune disease. They suggest that in contrast to Russian infants, Finnish infants mount an insufficient immune response due to exposure to *Bacteroides* LPS rather than *Escherichia coli* LPS. Utilization of *MetaLonDA* in this cohort demonstrates that Escherichia establishes a significant community in Russian infants from day 550 to 946 with little variability. *MetaLonDA* also defined specific time intervals during which other bacterial genera (e.g., *Lactobacillus*, *Leptotrichia*, *Klebsiella*) previously associated as protective or instigating of type 1 diabetes (T1D) were differentially abundant [33, 34]. Moreover, *MetaLonDA* established that up until day 629, Finnish infants present an additional shift in *Proteobacteria* with an overabundance of genera that are known to be implicated in human disease, including *Campylobacter*, *Haemophilus*, *Klebsiella*, and *Neisseria*. In parallel, when evaluating genera that have previously been associated with protection against T1D, *MetaLonDA* reveals a divergence from *Lactobacillus* and *Lactococcus* to *Veillonella* as the dominant *Firmicutes* genera observed early in the life of Finnish infants. These findings suggest that there is a complex interplay of multiple bacterial genera early in life which may all have immunogenic potential and will allow, in this case, further exploration of the role of bacteria-specific LPS as well as other microbial specific stimulators or inhibitors of the host immune response and their role in development of autoimmune disease.

## Conclusion

We have developed *MetaLonDA* as an R package that can identify significant time intervals of differentially abundant microbial features such as taxonomies, genes, or pathways. *MetaLonDA* is flexible such that it can perform differential abundance tests on longitudinal samples with different numbers of subjects per phenotypic group, different numbers of samples per subject, and samples that are not collected at consistent time points. These inconsistencies are often the case for samples collected from human subjects. Inconsistencies increase with the complexity of the procedure utilized to obtain the samples. Usually, there is less inconsistency in samples collected through non-invasive procedures such as stool and urine samples but increases in the case of invasive procedures such as BAL. *MetaLonDA* relies on two modeling components: the NB distribution for modeling the mapped read counts for each feature and the semi-parametric SS-ANOVA technique for modeling longitudinal profiles associated with different phenotypes.

Extensive experiments on simulated datasets quantitatively demonstrate the effectiveness of *MetaLonDA* with significant improvement over alternative methods. The time needed to execute *MetaLonDA* depends on the number of features being tested and the number of permutations for generating AR empirical distributions. *MetaLonDA* performs significance testing based on unit time intervals that can be hours, days, weeks, months, or years. The identified time intervals of differentially abundant features can be used as preselected features for a machine learning classifier to predict disease prognosis [35–37]. *MetaLonDA* can be applied to any longitudinal count data such as metagenomic sequencing, 16S rRNA gene sequencing, or RNA-Seq. It is worth noting that the NB assumption made for taxonomy would need to be reassessed before *MetaLonDA* can be confidently applied to functional data. In the future, we plan to implement a checker function that evaluates the distributional assumption based on KS test, and accordingly, the best fitted model can be utilized for the longitudinal differential abundance test.

Furthermore, *MetaLonDA* allows for an in-depth exploration of potential features and establishment of precise time intervals during which individual features may serve as biomarkers from population-based longitudinal studies such as the DIABIMMUNE cohort discussed in this paper. Specific significant time intervals can then be utilized to establish targeted timely screening or prevention of individual features and allow for prompt intervention, such as the use of antibiotics or probiotics. Unlike with cross-sectional methods that are incapable of identifying significant time intervals associated with differentially abundant features, *MetaLonDA* may lead to reconstitution of the microbiome and reestablish homeostasis prior to entering the cascade of events that may lead to overt disease.

Although *MetaLonDA* addresses one of the most common limitations in human sample collection inconsistencies, there is still room for improvement. The current version of *MetaLonDA* only finds the association between microbial features, time, and phenotypic group. In the future, we plan to incorporate additional confounding factors (age, gender, race, disease severity, etc.) to the *MetaLonDA* model. Another limitation of *MetaLonDA* is that when samples are sparse over extended time intervals, the fitted smoothing spline has large variation [16]. This causes the identified significant time intervals to be unreliable and should be excluded from the analysis. Thus, identification of these extended intervals based on a statistical method merits further investigation.

*MetaLonDA* is publicly available on the CRAN repository (https://CRAN.R-project.org/package=MetaLonDA).

## Availability and requirements

**Project name:** *MetaLonDA*

**Project home page:** https://CRAN.R-project.org/pack- age=MetaLonDA

**Source-code available at:** https://github.com/aametwa- lly/MetaLonDA

**Operating system(s):** Platform independent

**Programming language:** R (≥ 3.2.0)

**License:** MIT

## Additional files

Additional file 1 The mathematical derivation of *MetaLonDA* algorithm. (PDF 214 kb)

Additional file 2 Zero-inflation probability distribution of the fitted ZIP distribution. Read counts are taken from the Caporaso et al., study. (PDF 3369 kb)

Additional file 3 Time point distribution per subject in the DIABIMMUNE study. (PDF 6472 kb)

Additional file 4 Evaluation of suitability of using different parametric distribution to model genera read counts from the DIABIMMUNE study. (PDF 476 kb)

Additional file 5 Table of all details of the identified time intervals by *MetaLonDA*, *LOWESS*, and *MetaSplines* in the hygiene hypothesis study. (CSV 21 kb)

Additional file 6 The identified time intervals of the shared differentially abundant genera by *MetaLonDA*, *LOWESS*, and *MetaSplines* between Finnish and Russian infants. (PDF 869 kb)

Additional file 7 The identified time intervals of the differentially abundant genera by *MetaLonDA* between Finnish and Russian infants. (PDF 804 kb)

## Abbreviations

BAL: Bronchoalveolar lavage
BH: Benjamini-Hochberg
KS test: Kolmogorov-Smirnov test
LPS: Lipopolysaccharides
MetaLonDA: Metagenomic longitudinal differential abundant
MGS: Metagenomic shotgun
NB: Negative binomial
OTU: Operational taxonomic unit
SS-ANOVA: Smoothing spline ANOVA
T1D: Type 1 diabetes
ZIP: Zero-inflated poisson
