# Supplementary material for: MetaLonDA: a flexible R package for identifying time intervals of differentially abundant features in metagenomic longitudinal studies
Source: Microbiome. 2018 Feb 13;6:32. doi: 10.1186/s40168-018-0402-y (PMC5812052; doi:10.1186/s40168-018-0402-y)
Supplement: Supplementary file 1 — The mathematical derivation of MetaLonDA algorithm. (PDF 214 kb) [file 40168_2018_402_MOESM1_ESM.pdf]

MetaLonDA: a flexible R package for identifying time intervals of differentially abundant features in metagenomic longitudinal studies

Ahmed A. Metwally, Jie Yang, Christian Ascoli, Yang Dai†,
Patricia W. Finn†, and David L. Perkins†

Supplementary Material

1 The mathematical derivation of MetaLonDA algorithm

Fixing a feature $f = 1, \dots, F$, the data under consideration are the random variables Y_{tki} or their observations y_{tki} of mapped reads of the ith subject of phenotype k to the feature f at time point t, where $t = 1, \dots, T$, $k = 1, 2$, and subject $i = 1, \dots, n_k$.

The random variable Y_{tki} is assumed to follow a negative binomial distribution

$$Y_{tki} \sim NB(\alpha, p(t, k)) \tag{1}$$

with integer $\alpha > 0$ and success probability $p(t, k) \in (0, 1)$. That is, Y_{tki} stands for the number of failures before the αth success in a sequence of Bernoulli trials. Then the probability for observing y number of reads can be written as

$$P(Y_{tki} = y) = \frac{\Gamma(\alpha + y)}{y!\Gamma(\alpha)} \cdot p(t, k)^{\alpha} \cdot (1 - p(t, k))^{y} \tag{2}$$

with an expectation and variance

$$E(Y_{tki}) = \frac{\alpha(1 - p(t, k))}{p(t, k)} \tag{3}$$

$$Var(Y_{tki}) = \frac{\alpha(1 - p(t, k))}{p(t, k)^2} \tag{4}$$

To model the time and phenotypic effect we use a general linear model with a logit link:

$$\eta(t, k) = \log \frac{p(t, k)}{1 - p(t, k)} \tag{5}$$

From Eq. (5), we have

$$p(t, k) = \frac{e^{\eta(t,k)}}{1 + e^{\eta(t,k)}} \tag{6}$$

$$1 - p(t, k) = \frac{1}{1 + e^{\eta(t,k)}} \tag{7}$$

Assuming Y_{tki}'s are independent, the log likelihood given a time-course

metagenomic count profiles $\mathbf{y} = \{y_{tki}\}_{t=1,\ldots,T;k=1,2;i=1,\ldots,n_k}$ is calculated as:

$$\begin{aligned}
\mathcal{L} &= \log L(\mathbf{p}, \alpha \mid \mathbf{Y} = \mathbf{y}) \\
&= \sum_{t=1}^{T}\sum_{k=1}^{2}\sum_{i=1}^{n_k}[y_{tki}\log(1-p(t,k)) + \alpha \log p(t,k) \\
&\quad + \log\Gamma(\alpha + y_{tki}) - \log\Gamma(\alpha) - \log(y_{tki}!)] \\
&= \sum_{t=1}^{T}\sum_{k=1}^{2}\sum_{i=1}^{n_k}[y_{tki}\log(1-p(t,k)) + \alpha \log p(t,k) \\
&\quad + \log\Gamma(\alpha + y_{tki}) - \log\Gamma(\alpha)] + \text{constant} \qquad (8)
\end{aligned}$$

Given the success probabilities $\mathbf{p} = \{p(t,k)\}_{t=1,\ldots,T;k=1,2}$ or equivalently the linear predictors $\boldsymbol{\eta} = \{\eta(t,k)\}_{t=1,\ldots,T;k=1,2}$, the main part of $\mathcal{L}$ involving α is

$$\mathcal{L}_p(\alpha) = \sum_{t=1}^{T}\sum_{k=1}^{2}\sum_{i=1}^{n_k}[\log\Gamma(\alpha + y_{tki}) - \log\Gamma(\alpha) + \alpha \log p(t,k)] \qquad (9)$$

which will be maximized to update α later.

Given the number of failures $\alpha > 0$, using Eqs. (6), (7), (8), we have the main part of $\mathcal{L}$ involving $\mathbf{p}$ or $\boldsymbol{\eta}$:

$$\mathcal{L}_\alpha(\boldsymbol{\eta}) = \sum_{t=1}^{T}\sum_{k=1}^{2}\sum_{i=1}^{n_k}[\alpha\eta(t,k) - (\alpha + y_{tki})\log(1 + e^{\eta(t,k)})] \qquad (10)$$

We seek the estimation of model parameters α and $p(t,k)$ by solving the optimization minimization Eq. (8). Following (Gu, 2013) [1], in order to control the smoothness of the function η, a roughness penalty $J(\eta)$ is added to the minus log-likelihood together with the smoothing parameter $\lambda > 0$ for the trade-off between the goodness of fit and the smoothness of the spline curve:

$$\min_{p,\alpha} -\mathcal{L} + \lambda \cdot J(\eta) \qquad (11)$$

In the objective function, $\mathcal{L}$ encourages the goodness of fit; $J(\eta)$ quantifies the smoothness of η, which is essentially the inner product in a reproducing kernel Hilbert space (Gu, 2013) [1], Section 3.1). The λ in expression (11) controls the tradeoff between the goodness of fit and the smoothness of the spline and can be determined using performance-oriented iterations or cross-validation (Gu, 2013 [1] Section 5.2).

The solution to the optimization problem in Eq. (11) leads to the smoothing spline that fits the reads from the samples across multiple time points. With the estimated parameters α and $p(t,k)$, we obtain the estimated mean of Y_{tki} using Eqs. (3), (6), (7), i.e.,

$$E(\hat{Y}_{tki}) = \hat{\alpha}e^{\hat{\eta}(t,k)} = \frac{\hat{\alpha}\hat{p}(t,k)}{1-\hat{p}(t,k)} \qquad (12)$$

Connecting the values at each time point using Eq. (12) the fitted curve can be constructed in each group. With Eqs. (4) and (12), the confidence intervals can be obtained for each feature. We use the `R` package `gss` (Gu, 2013 [1]) to solve problem Eq. (11). For readers' reference, a more detailed description for the algorithm used in [1], Section 5.4.6) with a specified $\lambda > 0$ is given below:

0° Given data $\{y_{tki}\}_{t=1,\ldots,T;k=1,2;i=1,\ldots,n_k}$, find the maximum likelihood estimate for the usual logistic regression model with negative binomial responses. That is, determine $\tilde{\alpha}^{(0)}, \tilde{p}^{(0)}(t,k), t = 1,\ldots,T; k = 1,2$ that maximize $\mathcal{L}$ in Eq. (8). Denote

$$\tilde{y}_{tki}^{(0)} = y_{tki},\ \tilde{\eta}^{(0)}(t,k) = \log(\tilde{p}^{(0)}(t,k)/(1-\tilde{p}^{(0)}(t,k)))$$

$t = 1,\ldots,T; k = 1,2; i = 1,\ldots,n_k$.

For iteration $s = 1,\ldots,S$, do 1°, 2° and 3°:

1° Determine $\tilde{\alpha}^{(s)}$ that maximizes

$$\sum_{t=1}^{T}\sum_{k=1}^{2}\sum_{i=1}^{n_k}[\log\Gamma(\alpha+\tilde{y}_{tki}^{(s-1)}) - \log\Gamma(\alpha) + \alpha\log\tilde{p}^{(s-1)}(t,k)]$$

2° For $t = 1,\ldots,T; k = 1,2; i = 1,\ldots,n_k$, let

$$\begin{aligned}\tilde{u}_{tki}^{(s)} &= (\tilde{\alpha}^{(s)} + \tilde{y}_{tki}^{(s-1)})\tilde{p}^{(s-1)}(t,k) - \tilde{\alpha}^{(s)}\\ \tilde{w}_{tki}^{(s)} &= (\tilde{\alpha}^{(s)} + \tilde{y}_{tki}^{(s-1)})\tilde{p}^{(s-1)}(t,k)\cdot(1-\tilde{p}^{(s-1)}(t,k))\\ \tilde{y}_{tki}^{(s)} &= \tilde{\eta}^{(s-1)}(t,k) - \tilde{u}_{tki}^{(s)}/\tilde{w}_{tki}^{(s)}\end{aligned}$$

3° Use quasi-Newton approach to find $\tilde{\eta}^{(s)}(t,k)$'s that minimize the penalized weighted least squares functional

$$\frac{1}{T(n_1+n_2)}\sum_{t=1}^{T}\sum_{k=1}^{2}\sum_{i=1}^{n_k}\tilde{w}_{tki}^{(s)}(\tilde{y}_{tki}^{(s)} - \eta(t,k))^2 + \lambda J(\eta)$$

Let $\tilde{p}^{(s)}(t,k) = e^{\tilde{\eta}^{(s)}(t,k)}/(1+e^{\tilde{\eta}^{(s)}(t,k)})$, $t = 1,\ldots,T; k = 1,2$.

Once we have the two splines that fits each group's samples, we can then calculate the normalized area between the two curves for each unit time interval of the $T-1$ time intervals. The normalized Area Ratio (AR) is calculated as in Eq. (13), where $A_{t,t+1}^{k_1}$ and $A_{t,t+1}^{k_2}$ denote the area under the spline curve from time t to time $t+1$ for group 1 and group 2, respectively, $t = 1,\ldots,T-1$.

$$AR_{t,t+1} = \frac{A_{t,t+1}^{k_1} - A_{t,t+1}^{k_2}}{max(A_{t,t+1}^{k_1}, A_{t,t+1}^{k_2})} \qquad (13)$$

Then, we perform a permutation procedure by permuting the sample group labels to calculate the AR_b for the random samples for each time interval. The procedure is repeated B times. This is essential for calculating the p-value of each interval. The p_value is calculated using Eq. (14)

$$p\_value = \frac{\#(AR_b > AR)}{B} \qquad b = 1, ..., B \tag{14}$$

The significant time intervals are those with $p\_value < 0.05$ after multiple testing correction [2] which adjusts for the number of time intervals per feature and for the multiple features that are testing for.

2 Estimation of distributions parameters

For each vector of feature's reads counts, we used the `fitdistr` function from the *MASS* R-package [3] to estimate the parameters of each parametric distribution used in the paper except zero-inflated Poisson (ZIP) distribution. Here are the parameters for each distribution:

- Negative-binomial distribution: *size* and *mean*
- Poisson distribution: *lambda*
- Zero-inflated Poisson distribution: *p* and *lambda*
- Lognormal distribution: *mean* and *standard deviation*
- Normal distribution: *mean* and *standard deviation.*
- Exponential distribution: *rate*

Using the estimated parameters, we simulated N ($N = \#$ of samples of the Caporaso *et al.,* study [4]) random numbers are generated using the corresponding parametric distribution.

For zero-inflated Poisson distribution, we used the `zeroinfl` function from the *pscl* R-package [5, 6] to fit each features read counts with a ZIP. Then we extracted the p (zero-inflation probability) and the *lambda*. Using these parameters, we can generate N random numbers following ZIP with the estimated parameters using `rzipois` function from the *VGAM* R-package [7, 8]

3 Simulation

Longitudinal differentially abundant features are simulated with mean $\mu(t)$ which follows Eq. (15), where $\mathcal{N}$ denotes normal distribution, I denotes an indicator function, and $t = 0, \ldots, 20$.

$$
\begin{aligned}
\mu(t) =&\mathcal{N}(20,1) + [\mathcal{N}(20,1) * (5-t) * I(t<5)] + \\
&[2 * \mathcal{N}(20,1) * (t-8) * I(t>8 \& t \leqslant 11)] + \\
&[2 * \mathcal{N}(20,1) * (13-t) * I(t>11 \& t \leqslant 13)] + \\
&[\mathcal{N}(20,1) * (t-15) * I(t>15)]
\end{aligned} \tag{15}
$$

References

[1] Gu, C.: Smoothing spline ANOVA models (2013)

[2] Benjamini, Y., Hochberg, Y.: Controlling the False Discovery Rate: A Practical and Powerful Approach to Multiple Testing on JSTOR. Journal of the Royal Statistical Society **57**(1), 289–300 (1995)

[3] Venables, W.N., Ripley, B.D.: Modern Applied Statistics with S, 4th edn. Springer, New York (2002). http://www.stats.ox.ac.uk/pub/MASS4

[4] Caporaso, J.G., Lauber, C.L., Costello, E.K., Berg-Lyons, D., Gonzalez, A., Stombaugh, J., Knights, D., Gajer, P., Ravel, J., Fierer, N., Gordon, J.I., Knight, R.: Moving pictures of the human microbiome. Genome biology **12**(5), 50 (2011). doi:10.1186/gb-2011-12-5-r50

[5] Jackman, S.: pscl: Political Science Computational Laboratory (2015). http://pscl.stanford.edu/

[6] Zeileis, A., Kleiber, C., Jackman, S.: Regression Models for Count Data in R. Journal of Statistical Software **27**(8), 1–25 (2008). doi:10.18637/jss.v027.i08

[7] Yee, T.W.: Vector Generalized Linear and Additive Models. Springer Series in Statistics. Springer, New York, NY (2015). doi:10.1007/978-1-4939-2818-7. http://link.springer.com/10.1007/978-1-4939-2818-7

[8] Yee, T.W.: VGAM: Vector Generalized Linear and Additive Models (2015). http://CRAN.R-project.org/package=VGAM
