# Supplementary figures and images for: MetaLonDA: a flexible R package for identifying time intervals of differentially abundant features in metagenomic longitudinal studies
Source: Microbiome. 2018 Feb 13;6:32. doi: 10.1186/s40168-018-0402-y (PMC5812052; doi:10.1186/s40168-018-0402-y)

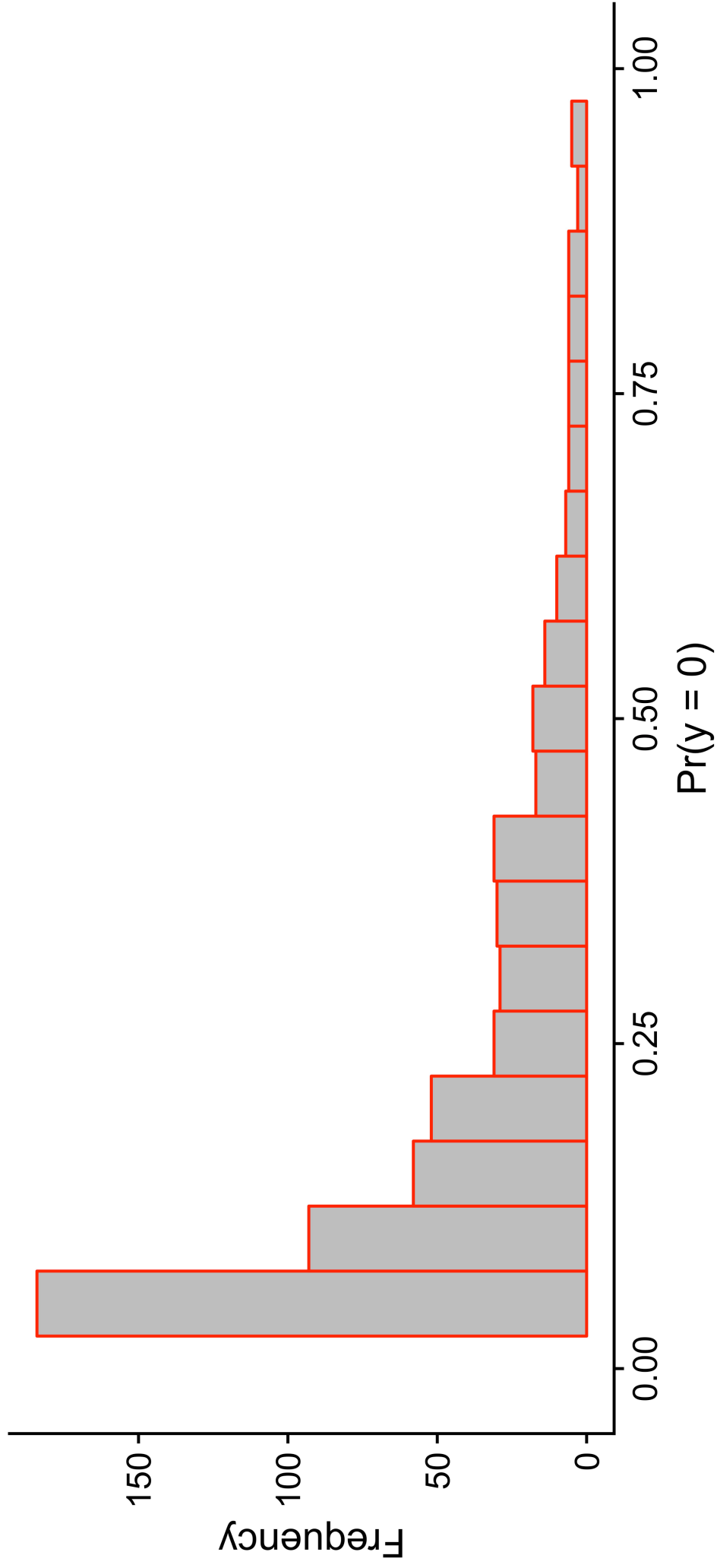

Supplement: Supplementary file 2 — Zero-inflation probability distribution of the fitted ZIP distribution. Read counts are taken from the Caporaso et al., study. (PDF 3369 kb) [file 40168_2018_402_MOESM2_ESM.pdf]

• FIN • RUS

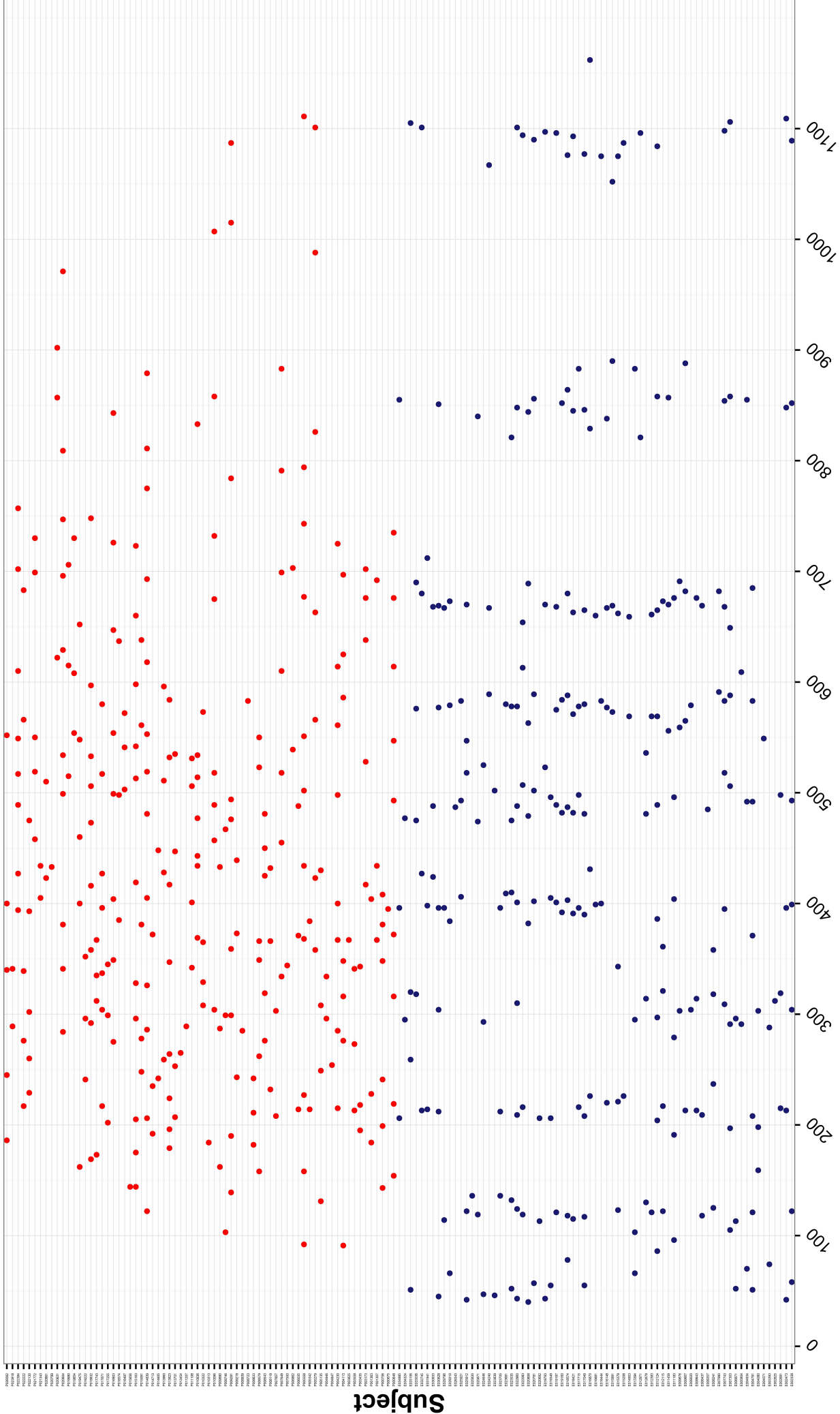

Supplement: Supplementary file 3 — Time point distribution per subject in the DIABIMMUNE study. (PDF 6472 kb) [file 40168_2018_402_MOESM3_ESM.pdf]

Method | MetaLonDA | LOWESS | MetaSplines

Dominant | FIN | RUS

Genus

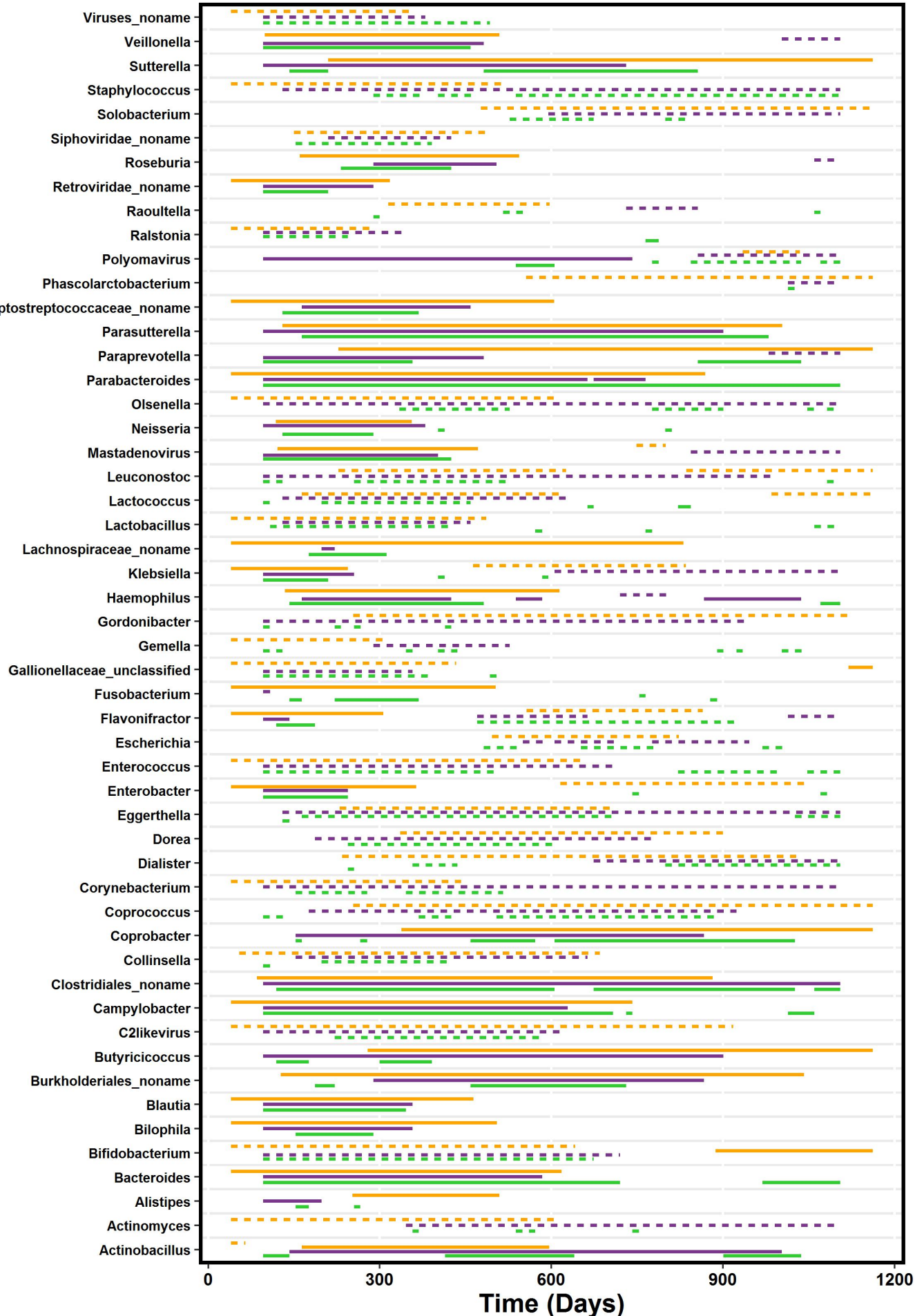

Supplement: Supplementary file 6 — The identified time intervals of the shared differentially abundant genera by MetaLonDA, LOWESS, and MetaSplines between Finnish and Russian infants. (PDF 869 kb) [file 40168_2018_402_MOESM6_ESM.pdf]

Dominant | FIN : RUS

Method | MetaLonDA

Genus

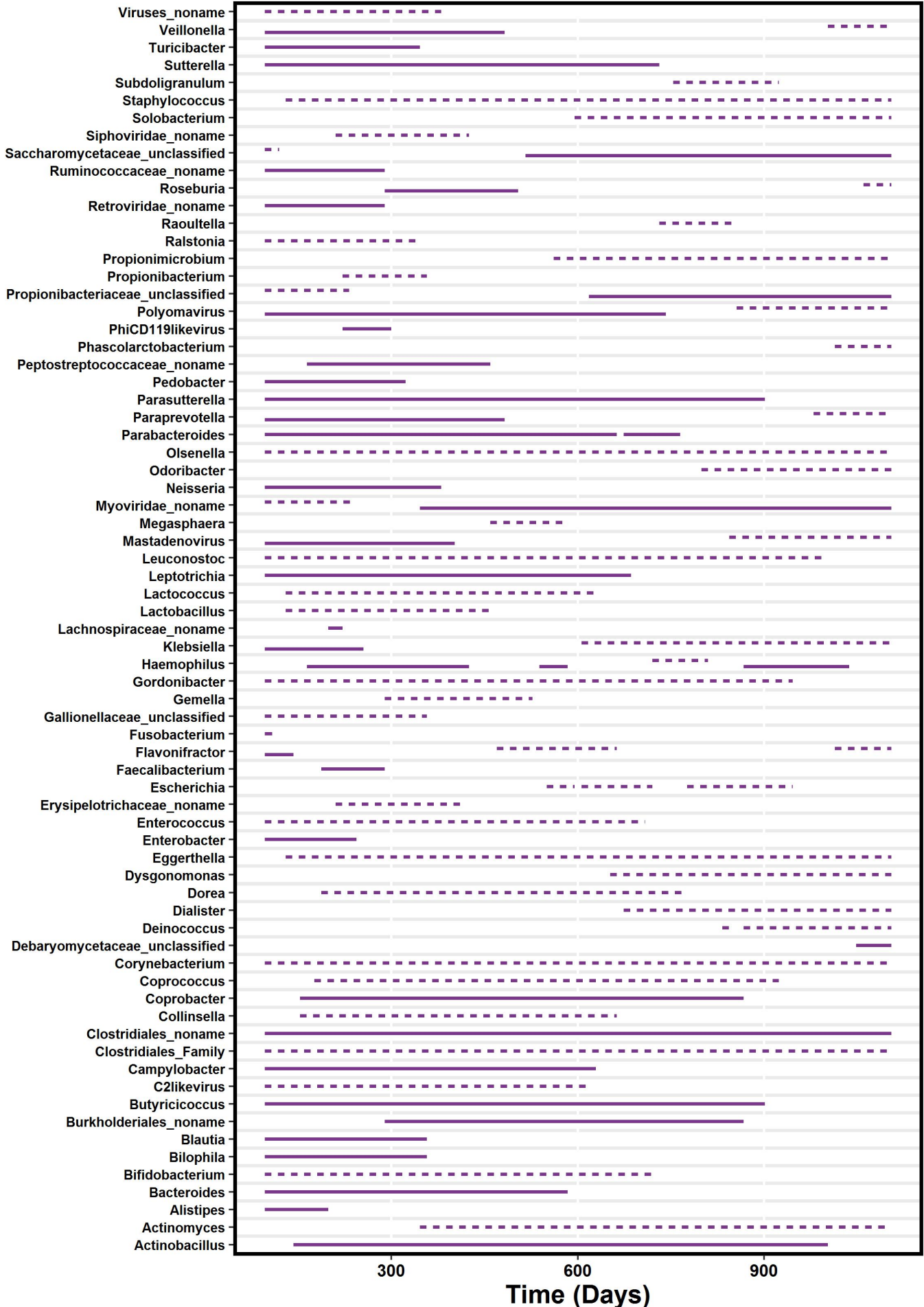

Supplement: Supplementary file 7 — The identified time intervals of the differentially abundant genera by MetaLonDA between Finnish and Russian infants. (PDF 804 kb) [file 40168_2018_402_MOESM7_ESM.pdf]
