# Supplementary material for: MetaLonDA: a flexible R package for identifying time intervals of differentially abundant features in metagenomic longitudinal studies
Source: Microbiome. 2018 Feb 13;6:32. doi: 10.1186/s40168-018-0402-y (PMC5812052; doi:10.1186/s40168-018-0402-y)
Supplement: Supplementary file 4 — Evaluation of suitability of using different parametric distribution to model genera read counts from the DIABIMMUNE study. (PDF 476 kb) [file 40168_2018_402_MOESM4_ESM.pdf]

Table S1: Number and percentage of genera out of 128 genera that do not show significant differences (p-value>0.05) with various standard statistical parametric distributions. The count data is taken from DIABIMMUNE study.

	#	%
**NB**	101	78.9
**ZIP**	46	35.9
**Poisson**	63	49.2
**Log-normal**	0	0
**Exponential**	0	0
**Half-normal**	0	0
**Normal**	0	0
